# Maternal high-fat diet during pregnancy with concurrent phthalate exposure leads to abnormal placentation

**DOI:** 10.1038/s41598-021-95898-4

**Published:** 2021-08-16

**Authors:** Athilakshmi Kannan, Juanmahel Davila, Liying Gao, Saniya Rattan, Jodi A. Flaws, Milan K. Bagchi, Indrani C. Bagchi

**Affiliations:** 1 Department of Comparative Biosciences, University of Illinois at Urbana-Champaign, Urbana, IL USA; 2 Department of Molecular and Integrative Physiology, University of Illinois at Urbana-Champaign, Urbana, IL USA

**Keywords:** Environmental sciences, Endocrinology

## Abstract

Di(2-ethylhexyl) phthalate (DEHP) is a synthetic chemical commonly used for its plasticizing capabilities. Because of the extensive production and use of DEHP, humans are exposed to this chemical daily. Diet is a significant exposure pathway and fatty food contain the highest level of phthalates. The impact on pregnancy following DEHP exposure and the associated interaction of high fat (HF) diet remains unknown. Here we report that exposure of pregnant mice to an environmentally relevant level of DEHP did not affect pregnancy. In contrast, mice fed a HF diet during gestation and exposed to the same level of DEHP display marked impairment in placental development, resulting in poor pregnancy outcomes. Our study further reveals that DEHP exposure combined with a HF diet interfere with the signaling pathway controlled by nuclear receptor PPARγ to adversely affect differentiation of trophoblast cells, leading to compromised vascularization and glucose transport in the placenta. Collectively, these findings demonstrate that maternal diet during pregnancy is a critical factor that determines whether exposure to an environmental toxicant results in impaired placental and fetal development, causing intrauterine growth restriction, fetal morbidity, and mortality.

## Introduction

Phthalates are synthetic chemicals widely used as plasticizers and stabilizers in various consumer products including shower curtains, children’s toys, cosmetics, and personal care products such as perfumes, nail polish, deodorants, and lotions^1,2^. Phthalates are also used in pesticides, wood finishes, adhesives, solvents, lubricants, defoaming agents, and medical devices, including tubing, blood bags, surgical gloves, and dialysis equipment^2^. An enormous amount of phthalates are used worldwide each year, and through inhalation, ingestion, and dermal contact, humans are exposed to this chemical in the range of 3 to 30 μg/kg/day^2^. Recent reports indicate that phthalate metabolites are present in nearly 100% of tested human urine samples^2–6^. Interestingly, phthalate metabolite levels are higher in women than men. This is likely due to higher use of personal care products by women compared to men^7^. Di2-ethylhexyl phthalate (DEHP) is one of the most abundant phthalates in the environment and is a principal contaminant in human tissue^1,2^. Increasing scientific evidence links phthalate exposure to harmful health outcomes due to endocrine-disrupting properties displayed by this class of chemicals. For example, chronic phthalate exposure is associated with decreased pregnancy rates, high miscarriage rates, anemia, toxemia, preeclampsia, and early menopause in women^1,2,8,9^. Phthalate exposures are also associated with adverse pregnancy outcomes including low birth weight and impaired childhood intellectual development and growth^10–13^.

Environmental exposures, particularly during pregnancy, can have long-lasting health impacts^14–16^. The uterine environment during pregnancy is a particularly important period of susceptibility; the trophoblast cells of the embryo, which give rise to the placenta, can be altered by chemical exposures, leading to abnormal placentation. During pregnancy in the mouse, as embryonic development proceeds, the trophoblast cells undergo differentiation and, along with fetal blood vessels, form extensive villous branching to create a densely packed structure called the labyrinth^17,18^. While the labyrinth is developing, syncytiotrophoblast cells remain in direct apposition to the endothelial cells of the fetal-derived blood vessels^17,19^. The labyrinth is supported structurally by spongiotrophoblast cells, which form a junctional zone between the labyrinth and the outer trophoblast giant cells^17,19^. Generation of these distinct trophoblast cell types within the placenta is necessary to accomplish the complex physiological processes of maternal–fetal exchange. Since a fully functional placenta is required to maintain pregnancy and support fetal development, any environmental insult that affects the placenta or its component lineages at any stage of development can lead to placental disorders^20–23^.

Only a limited number of studies address the impact of environmental exposure to phthalates on pregnancy. While Zong et al*.* showed that exposure to DEHP reduces embryo implantation, reduces placental growth, and increases embryonic loss in mice, these studies were performed using high doses that far exceeded the levels of environmental exposure^24^. A major route for exposure to phthalates is by diet^25^. Foods high in fat are contaminated by higher-weight phthalates such as DEHP^26^. Therefore, DEHP exposure in the context of a high-fat (HF) diet presents health concerns and the effect of combining these exposures on pregnancy outcome should be determined. Thus, in the present study, pregnant mice were exposed to an environmentally relevant level of DEHP and were fed a HF diet. We then analyzed how these exposures affected placental development and pregnancy outcome. Our results indicated that the combination of a HF diet and DEHP exposure causes disorganization of the trophoblast layers in the placenta and abnormal vascular patterning, indicating defective placental development. Our studies also revealed that DEHP exposure in the context of a HF diet interferes with the peroxisome proliferator-activated receptor (PPAR) γ signaling pathway, severely affecting its role in placenta development during pregnancy and causing fetal morbidity and mortality.

## Materials and methods

### Animals

The study is in compliance with ARRIVE guidelines for in vivo studies carried out on animals and all experiments involving animals were conducted in accordance with the National Institutes of Health standards for the use and care of animals. The animal protocols were approved by the University of Illinois Institutional Animal Care and Use Committee. CD-1 female mice of 7–8 weeks were purchased from Charles River and housed at 25 °C in ventilated cages on 12L:12D cycles. Rodent regular diet and high fat (HF) diet were purchased from Research Diet Inc. (Control diet has 20 g while HF has 177.5 g of fat; catalog numbers D12450B and D12451, respectively). At eight weeks of age, female mice were randomly separated into four groups: normal diet with corn oil, normal diet with 20 µg/kg body weight DEHP in corn oil, HF diet with corn oil, and HF diet with 20 µg/kg body weight DEHP in corn oil. These female mice were exposed to their corresponding diets for one week before they were mated with male mice fed with normal diet. The female mice were monitored for the presence of a copulatory vaginal plug to confirm mating and the presence of a vaginal plug was considered day 1 (D1) of pregnancy. Pregnant mice were weighed and orally dosed with corn oil or with 20 µg/kg body weight DEHP. The doses were calculated and adjusted based on daily body weights. Mice were dosed daily from D1 until euthanasia (D13, D16 or PND1).

### Tissue collection and analysis

Placentas were harvested from pregnant females on D13 and D16, and weighed. Fetuses were separated on D13 and D16 and weighed individually. After delivery, PND1 pups were collected and weighed. Parts of the placental tissues were processed for RNA isolation and the other parts were fixed in 10% neutral-buffered formalin. Placentas were embedded in paraffin, sectioned, and stained with hematoxylin and eosin or periodic acid-Schiff hematoxylin (PASH). Placental sections were also subjected to immunohistochemistry using the primary antibodies: proliferating cell nuclear antigen (PCNA, 1: 200, Santa Cruz Biotechnology, SC-56), cytokeratin 8 (KRT8, 1:50, Developmental Studies Hybridoma Bank, TROMA-1), trophoblast specific protein alpha, (1:500, Abcam, ab104401), platelet/endothelial cell adhesion molecule 1 (PECAM1/CD31, 1:250, Abcam, ab124432), pregnancy-associated plasma protein A (PAPP-A, 1:200, Bioss Antibodies, bs-6618r), p57^Kip2^ (1:100, Santa Cruz Biotechnology, SC-8298), Glucose transporter 1(GLUT1, 1:500, Abcam, ab128033), and Muc1 (1:200, Novus Biologicals, NB120-15,481). Secondary antibodies including fluorescent-tagged rhodamine donkey anti-rabbit, 488 donkey anti-mouse, 488 donkey anti-rabbit, 488 donkey anti-goat were purchased from Jackson Immuno Research. Fluoromount-G with DAPI was purchased from eBiosciences. Quantitation of immunofluorescence intensity was measured using ImageJ software v1.52v as described previously^27,28^.

Total RNA was extracted from placentas, reverse transcribed into cDNA, and used for quantitative PCR using the SYBR Green PCR mix in ABI thermal cycler 7500. Primers used are: *Gcm1: forward:* 5′-AACACCAACAACCACAACTCC-3′, reverse: 5′-CAGCTTTTCCTCTGCTGCTT-3′, *SynA: forward:* 5′-CCCTTGTTCCTCTGCCTACTC-3′, reverse: 5′-TCATGGGTGTCTCTGTCCAA-3′, *Muc1: forward:* 5′-TTGGTTGCTTTGGCTATCGT-3′, reverse: 5′-TTACCTGCCGAAACCTCCTC-3′, *Ctsq: forward:* 5′-AACTAAAGGCCCCATTGCTAC-3′, reverse: 5′-CAATCCCCATCGTCTACCC-3′, *Tpbpa: forward:* 5′-CGGAAGGCTCCAACATAGAA-3′, reverse: 5′-CGGAAGGCTCCAACATAGAA-3′, *Pparγ: forward:* 5′-CCACCAACTTCGGAATCAGCT-3′, reverse-5′CCGGCAGTTAAGATCACACCTAT-3′, *36B4: forward*: 5′-CATCACCACGAAAATCTCCA-3′ reverse-5′-TTGTCAAACACCTGCTGGAT-3′. The results were analyzed using the ABI Prism 7500 software (Applied Biosystems, USA). Gene expressions were normalized to housekeeping gene *Rplp0* (*36B4*). The fold change of gene expression in each sample relative to control was generated using the 2^−ΔΔCt^ mathematical model for relative quantification of quantitative PCR. Steroid hormones such as estrogen and progesterone were measured using enzyme-linked immunosorbent assays (DRG International Inc., New Jersey) according to the manufacturer’s protocol.

### Statistical analyses

Statistical analyses were performed as we have done previously^29^. Briefly, placental and pup weights, gene expression data, hormonal profiles, and relative intensities were expressed as mean ± S.E.M. Statistical analysis was done using a two-tailed Student’s *t-test*. One-way ANOVA with a Tukey post hoc test was performed in studies involving multiple comparisons. An analysis of equal variances was done on all numerical data to determine whether a parametric or non-parametric hypothesis test was appropriate. Survival curves were used to compare pup mortality by treatment groups. Data were considered statistically significant at p ≤ 0.05 and are indicated by asterisks in the figures. All data were analyzed and plotted using GraphPad Prism 9.0 (GraphPad Software).

## Results

### Combined exposure to DEHP and a HF diet during pregnancy affects fetal growth and mortality in mice

**Figure 1:**
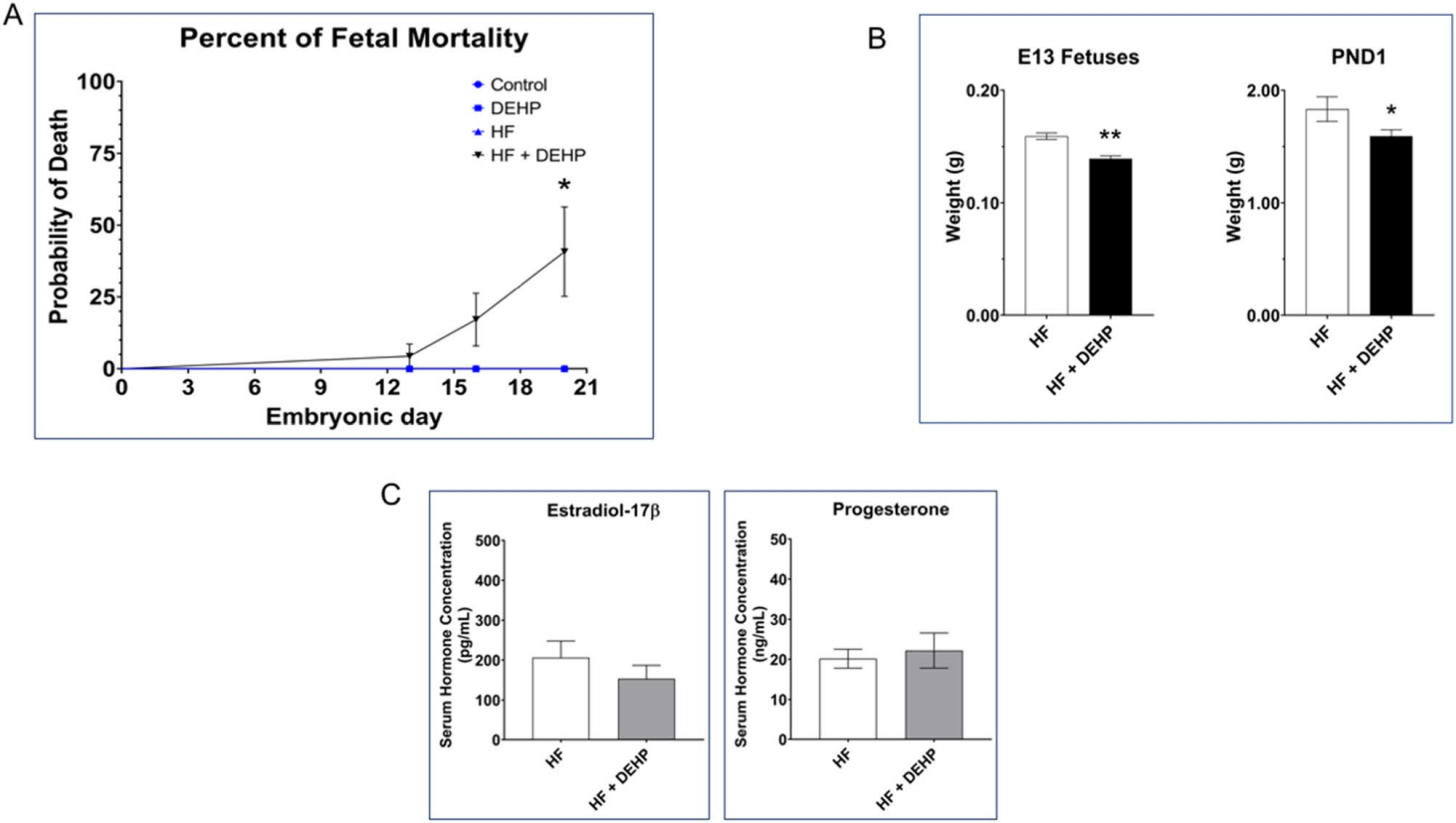
Exposure to DEHP and a HF diet during pregnancy affects fetal growth and mortality. (**A**) Female mice from day 1 of pregnancy were fed normal diet (control) or normal diet and exposed to 20 μg/kg/day of DEHP (DEHP), HF diet (HF) or HF diet and exposed to 20 μg/kg/day of DEHP (HF + DEHP) as described in the Materials and Methods. Fetal mortality was determined on D13 of pregnancy, D16 of pregnancy, and at the end of gestation. N = 8 /pregnancy day and the data are represented as the mean of pups pooled from 8 animals in each group. (**B**) Fetal weights were measured on D13 and postnatal day 1. N = 8 /pregnancy day and the data are represented as the mean of pups pooled from 8 animals in each group. (**C**) Serum hormone levels of progesterone and estrogen were determined in HF and DEHP + HF animals on D16 of gestation. Asterisks indicate statistically significant differences *p < 0.05, **p  < 0.001.

Humans are usually exposed to 3–30 µg/kg of DEHP daily^2^. According to the Environmental Protection Agency, 20 µg/kg/day DEHP exposure is considered a safe exposure limit. A previous study using mice showed that exposure to a high dose of DEHP (500 mg/kg/day) is detrimental to pregnancy^24^. This report raised the question whether an environmentally relevant low exposure to DEHP would impact the pregnancy outcome under certain metabolic or dietary conditions. Since fatty food contain the highest level of phthalates, it raises the possibility that exposure to even a low dose of DEHP in the context of HF diet may impact gestation. To test this hypothesis, we designed a study in which pregnant mice under normal and HF dietary conditions were exposed to 20 µg/kg/day DEHP and then determined the effect of these exposures on pregnancy outcomes. Mice fed (i) normal diet (control), (ii) normal diet with exposure to DEHP (DEHP), (iii) a HF diet without exposure to DEHP (HF), and (iv) a HF diet with exposure to DEHP (HF + DEHP) were analyzed for the number of implanted embryos on day 13 (D13), fetuses on day 16 (D16) and post-natal day 1 (PND1). Our results showed that females exposed to an environmentally relevant level of DEHP and fed a HF diet (HF + DEHP) delivered significantly fewer live pups compared to mice (i) fed normal diet (control), (ii) fed normal diet with exposure to DEHP (DEHP), and (iii) fed a HF diet without exposure to DEHP (HF) (Fig. 1A). No significant difference in the number of implantation sites on D13 or D16 (data not shown) or litter size (live and dead pups) was observed among the different treatment groups (Supplementary Fig. S1). While there were no fetal deaths in control, DEHP or HF exposed mice, we observed a significant increase in pup mortality in the HF + DEHP group. The deaths of the pups occurred in utero between days 18 to 20 of gestation. Collection of fetuses and placentas from mid-gestation to late pregnancy revealed decreased fetal weights in HF + DEHP dams on D13 of pregnancy and PND1 compared to HF dams (Fig. 1B). The placental weights were not affected in any group (data not shown). These impairments in pregnancy outcome and fetal growth were not due to altered levels of progesterone and estrogen. As shown in Fig. 1C, serum levels of these hormones did not differ significantly between the HF and HF + DEHP animals during mid-gestation. Collectively, these results indicated that DEHP exposure in combination with a HF diet during pregnancy affects fetal growth and mortality.

### Pregnant mice fed a HF diet and exposed to DEHP exhibit altered placental architecture

**Figure 2:**
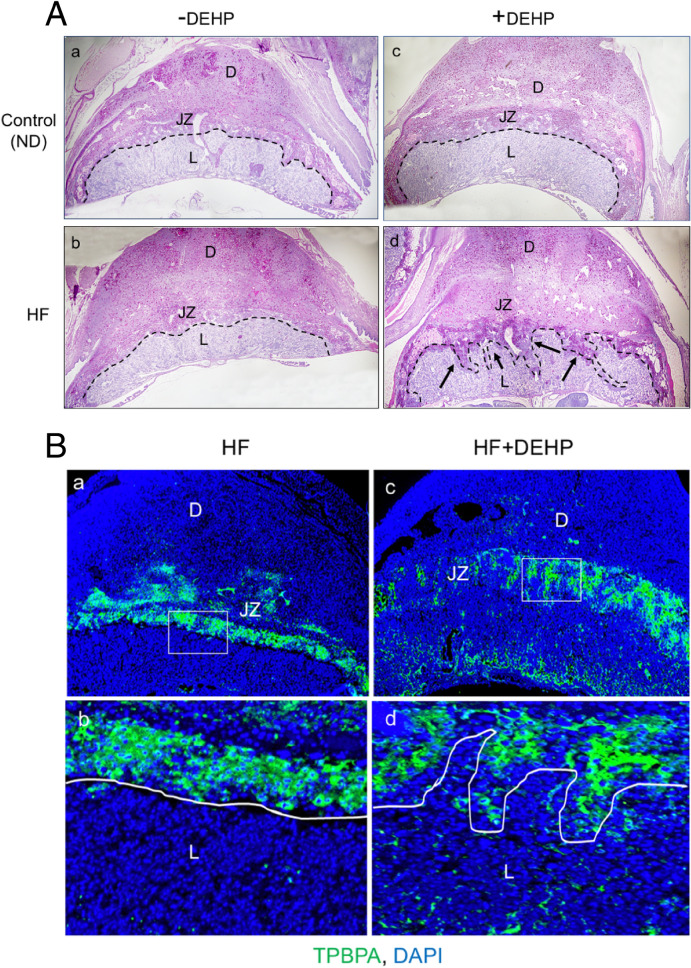
Placental architecture is altered in mice exposed to DEHP and fed a HF diet. (**A**) Periodic Acid-Schiff Hematoxylin (PASH) staining of control, which is normal diet or ND (**a**), HF (**b**), DEHP (**c**), and HF + DEHP (**d**) placental sections on D13 of pregnancy are shown (4×). Black arrows indicate the trophoblast cells from the junctional zone that have invaded the labyrinth. L: labyrinth, JZ: junctional zone, D: decidua. N = 8 for each group and representative images are shown. (**B**) Placental sections from HF (panels **a** and **b**) and DEHP + HF (panels **c** and **d**) mice on D13 of pregnancy were subjected to immunofluorescence using trophoblast specific protein alpha (TPBPA) antibody. TPBPA positive spongiotrophoblast layer is demarcated by a white line. L: labyrinth, JZ: junctional zone, D: decidua. N = 5 for each group and representative images are shown.

To investigate the cause of fetal growth restriction and mortality due to DEHP exposure and a HF diet, we analyzed placental morphology employing Periodic Acid-Schiff Hematoxylin staining (PASH) on D13 of pregnancy. PASH detects polysaccharides including glycogen. Since placental sex is a major determinant in the functional response of the placenta during pregnancy, we determined placental sex by PCR using primers specific for *Sry and Il3* genes as previously described^30^. In this study we focused exclusively on female placentas. As expected, placentas of pregnant mice under normal (control) or HF dietary conditions on D13 consists of three main layers: the labyrinth, the junctional zone consisting of spongiotrophoblast and glycogen-rich trophoblast cells, and a layer of parietal trophoblast giant cells bordering the maternally derived decidua (Fig. 2A, panels a,b). Similar placental architecture was observed in mice under normal dietary conditions exposed to 20 µg/kg/day DEHP (Fig. 2A, panel c). Interestingly, we found marked impairment in placental development when mice fed a HF diet were exposed to the same low level of DEHP during pregnancy. As shown in Fig. 2A, panel D, the junctional zone was disorganized, with spongiotrophoblast and glycogen-rich trophoblast cells having infiltrated the labyrinth layer. To further analyze this abnormal placental morphology, placental samples were obtained from four experimental groups fed (i) normal diet (control), (ii) normal diet with exposure to DEHP (DEHP), (iii) HF diet without exposure to DEHP (HF), and (iv) HF diet with exposure to DEHP (DEHP + HF) and were probed for trophoblast-specific protein alpha (TPBPA) using immunofluorescence. In mice fed normal diet (control) or normal diet with exposure to DEHP (DEHP), the TPBPA-positive spongiotrophoblast cells were localized exclusively in the junctional zone while none were detected in the labyrinth layer (Supplementary Fig. S2). The TPBPA-positive spongiotrophoblast cells were also localized exclusively in the junctional zone in mice fed HF diet (Fig. 2B, panels a,b). In contrast, TPBPA immunostaining revealed that the labyrinth layer in DEHP + HF placentas showed aberrant infiltration by spongiotrophoblast cells (Fig. 2B, panels c,d), indicating impaired placental development.

### DEHP exposure of pregnant mice fed a HF diet affects trophoblast differentiation

**Figure 3:**
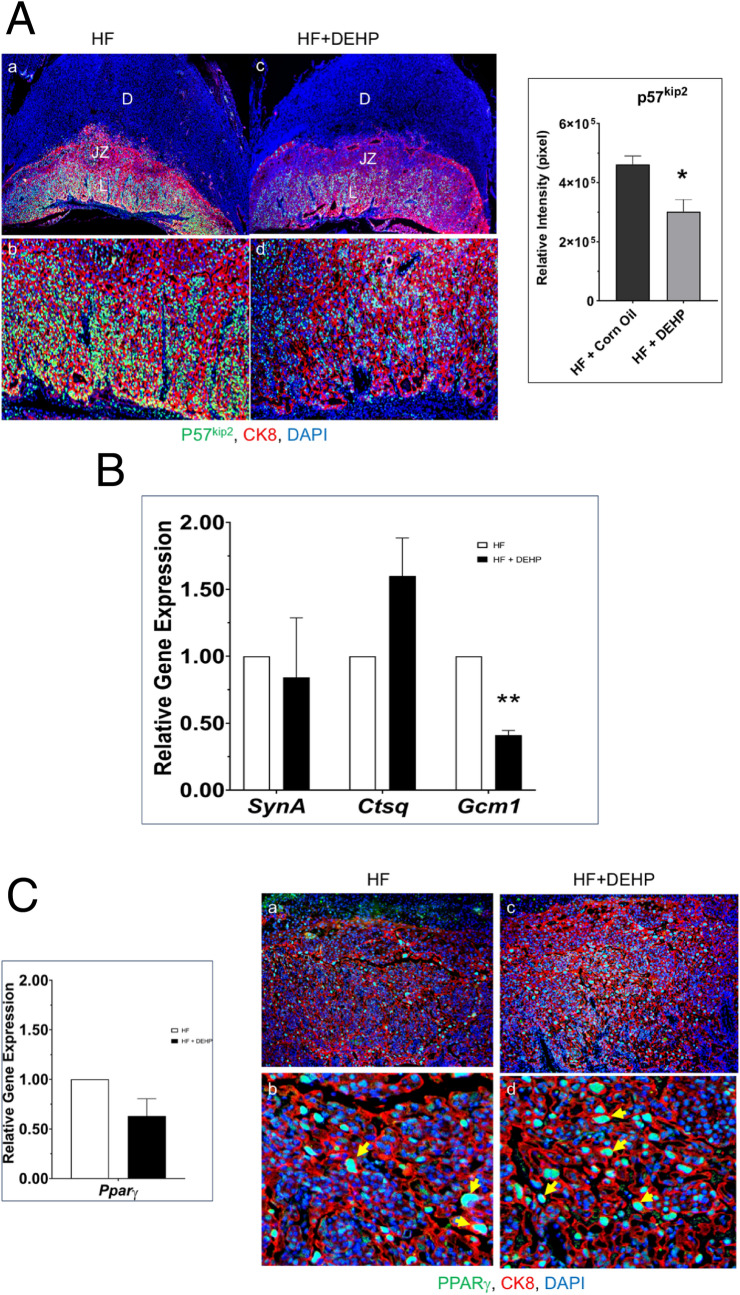
DEHP exposure and HF diet affects trophoblast differentiation. (**A**) Left. Placental sections from HF (panels **a** and **b**) and HF + DEHP (panels **c** and **d**) mice on D13 of pregnancy were subjected to immunofluorescence using P57^Kip2^ and cytokeratin 8 antibodies. Panels (**a** and **c**): 4X magnification. Panel (**b**) represents a magnified image (10×) of the labyrinth layer of HF placentas while panel d represents a magnified image (10×) of the labyrinth layer of HF + DEHP placentas. Green indicates immunostaining with P57^kip2^. Red indicates immunostaining with cytokeratin 8 and blue indicates DAPI. L: labyrinth, JZ: junctional zone, D: decidua. N = 5 for each group and representative images are shown. Right. ImageJ analysis of P57^Kip2^ positive cells. The values represent mean ± SEM of five independent samples. Asterisks indicate statistically significant differences *p < 0.05. (**B**) Comparable expressions of various factors involved in trophoblast differentiation in placentas from HF and HF + DEHP animals. Total RNA was isolated from the placenta on D13 of pregnancy and qPCR analysis was performed using primers specific for *Ctsq*, *Gcm1*, and *SynA*. Data represent mean ± SEM from four separate samples. Asterisks indicate statistically significant differences **p < 0.001. (**C**) Left. Expression of *PPARγ* in placentas from HF and HF + DEHP animals. Total RNA was isolated from the placenta on D13 of pregnancy and qPCR analysis was performed using primers specific for *PPARγ*. Data represent mean ± SEM from four separate samples. Right. Placental sections from HF (panels **a** and **b**) and HF + DEHP (panels **c** and **d**) mice on D13 of pregnancy were subjected to immunofluorescence using PPAR*γ* and cytokeratin 8 antibodies. Panel (**b**) (20×) represents a magnified image of the labyrinth layer of HF placentas while panel d (20×) represents a magnified image of the labyrinth layer of HF + DEHP placentas (10×). Green indicates immunostaining with PPAR*γ*. Red indicates immunostaining with cytokeratin 8 and blue indicates DAPI. Yellow arrows indicate nuclear localization of PPAR*γ*. N = 5 for each group and representative images are shown.

Trophoblast differentiation is a critical step in the process of establishing placental lineages that govern placental development, maintenance, and function. P57^Kip2^, a cyclin-dependent kinase, plays an important role in the development of labyrinth trophoblasts and spongiotrophoblasts in both human and mouse placentas^31^. To investigate whether trophoblast differentiation is affected by DEHP exposure of mice fed a HF diet, we used immunofluorescence to monitor the expression of P57^Kip2^ in placental samples on D13 of pregnancy. Placentas of mice fed a normal diet or normal diet with exposure to DEHP or HF diet displayed comparable abundant P57^Kip2^ expression in the junctional zone and labyrinth layer of placentas, indicating normal development of trophoblast cells (Fig. 3A, left, and Supplementary Fig. S3). However, a marked decline in the expression of P57^Kip2^ was observed in placentas of DEHP + HF mice (Fig. 3A, left). To quantify the immunohistochemical findings, ImageJ analysis was used as described previously^27,28^. Our analysis revealed a significant decline of P57^Kip2^ immunostaining in HF + DEHP placentas compared to HF placentas (Fig. 3A, right, *p < 0.05). Collectively, these results indicate that exposure to HF + DEHP leads to compromised trophoblast differentiation.

We then investigated the combined impact of DEHP and a HF diet on the development of specific trophoblast subtypes, syncytiotrophoblast cells (SynT-I and SynT-II) and the sinusoidal trophoblast giant cells, which are major constituents of the labyrinth^17,32,33^. As shown in Fig. 3B, the expressions of SynA, a specific marker for SynT-I cells, and Ctsq, a specific marker for sinusoidal trophoblast giant cells, were not significantly altered in HF + DEHP placentas compared to HF placentas, indicating that the development of these cells were not affected by DEHP treatment. Interestingly, we found a marked decline in the expression of glial cell missing-1 (Gcm1), a transcription factor known to play a critical role in the differentiation of SynT-II syncytiotrophoblast cells of the labyrinth^34^ (Fig. 3B). Taken together, these results indicated that DEHP treatment combined with a HF diet downregulates the expression of Gcm1 in placentas and as a consequence impacts the differentiation of SynT-II cells of the labyrinth layer.

Previous studies have shown that transcription factor peroxisome proliferator-activated receptor gamma (PPAR*γ*) regulates Gcm1 expression^35,36^ and placental development^37–39^. PPARγ null mice showed 100% embryonic lethality due to defects in differentiation of labyrinth trophoblasts^37–39^. Consistent with this observation, a deficiency of PPARγ in mouse trophoblast stem cells was shown to affect the differentiation of labyrinth cell lineages with a concurrent decrease in Gcm1^35^. We therefore investigated whether the placental expression of PPAR*γ* is altered in HF + DEHP mice. As shown in Fig. 3C, the PPARγ mRNA levels in the placenta were not altered when pregnant HF mice were dosed with DEHP (left panel). We also performed immunohistochemical analysis to investigate PPARγ protein expression and found PPARγ protein levels were comparable in the placentas of HF + DEHP and HF mice (Fig. 3C, right panel). Taken together, these results indicated that exposure to DEHP does not alter PPARγ expression in placentas.

### Combined exposure to DEHP and a HF diet suppresses expression of downstream targets of PPARγ

We next examined whether DEHP treatment alters the function of PPAR*γ* thereby affecting its downstream signaling pathways in the placenta. We analyzed the expression of pregnancy-associated plasma protein-A (PAPP-A) and mucin (MUC1), which are known to be regulated by PPARγ in trophoblast cells during placenta development^40^. PAPP-A is a secreted metalloproteinase, which cleaves insulin-like growth factor binding proteins (IGFBPs) to regulate the bioavailability of local insulin-like growth factors (IGFs) which in turn control trophoblast proliferation and differentiation^41^. Muc1 is a glycoprotein, which is localized exclusively to the apical surface of the placental labyrinthine trophoblast around maternal blood sinuses^40^.

**Figure 4:**
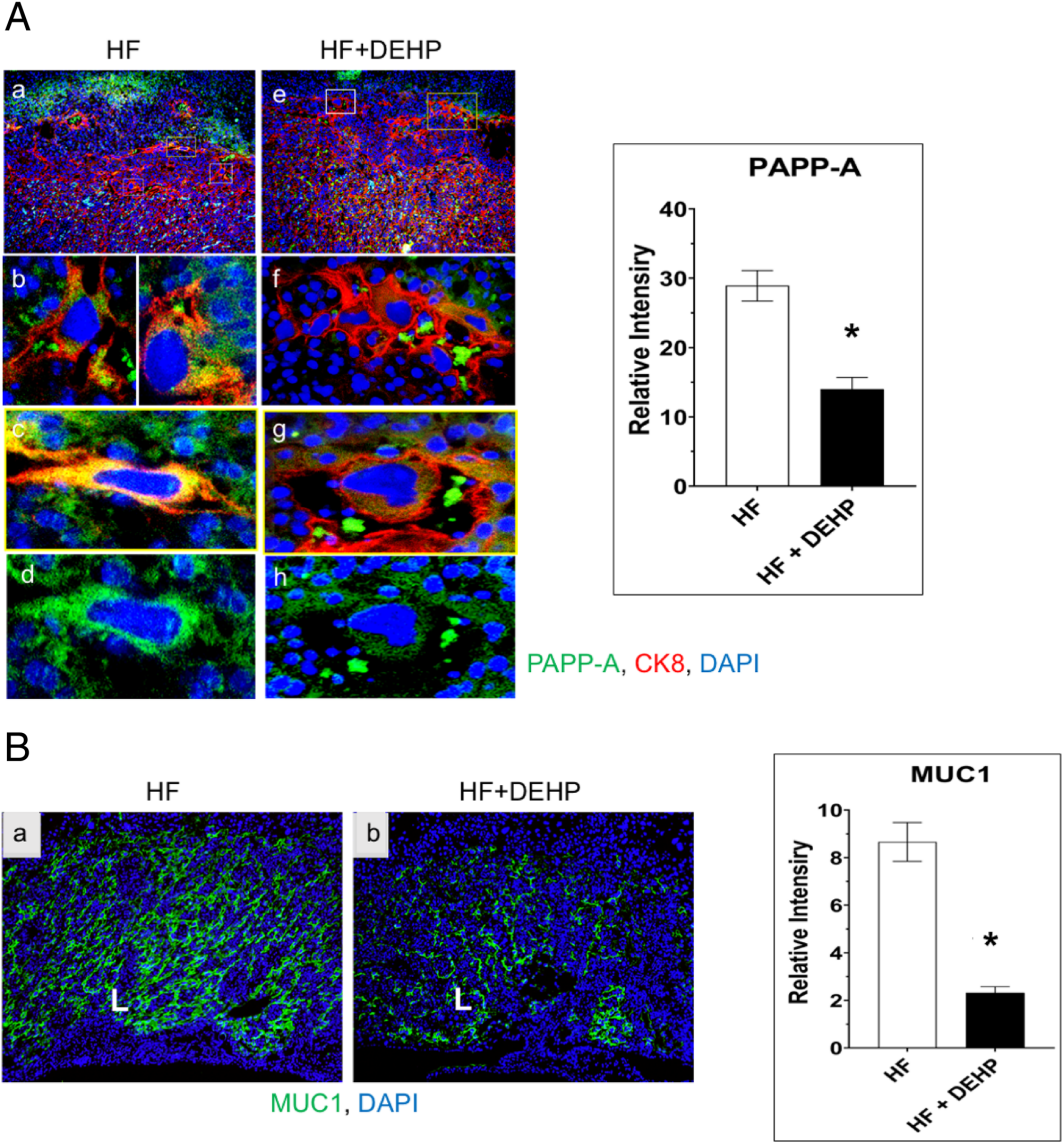
Exposure to DEHP and a HF diet attenuates expression of downstream targets of PPARγ. (**A**) Left. Placental sections from HF (panels **a**, **b**, **c**, and **d**) and HF + DEHP (panels **e**, **f**, **g**, and **h**) mice on D13 of pregnancy were subjected to immunofluorescence using PAPP-A (green) and cytokeratin 8 (red) antibodies. Note co-localization of cytokeratin 8 and PAPP-A (yellow) in the invading trophoblast cells at the border of the junctional zone and decidua of HF placentas, indicated by the boxed areas in panel (**a**). Panels (**b** and **c**) represent magnified images of trophoblast cells at the border of the junctional zone and decidua of HF placentas (4×) with co-localization of cytokeratin 8 and PAPP-A. Panel (**d**) shows trophoblast cells with PAPP-A but no cytokeratin 8 staining. In HF + DEHP placentas, the expression of PAPP-A was attenuated in the invading trophoblast cells at the border of the junctional zone and decidua, indicated by the boxed areas in panel (**e**). Panels (**f** and **g**) represent magnified images of trophoblast cells at the border of the junctional zone and decidua of HF + DEHP placentas (4×) with co-localization of cytokeratin 8 and PAPP-A. Panel (**h**) shows trophoblast cells with PAPP-A but no cytokeratin 8 staining. Right. Immuno-positive cells for PAPP-A were analyzed by ImageJ software. The values represent mean ± SEM of five independent samples. (**B**) MUC1 immunostaining of placental labyrinth from HF (panel **a**) and HF + DEHP (**b**) animals on D13 of pregnancy (10×). Green indicates immunostaining with MUC1 and blue indicates DAPI. Right. Immuno-positive cells for MUC1 were analyzed by ImageJ software. The values represent mean ± SEM of five independent samples. Asterisks indicate statistically significant differences *p < 0.05.

As shown in Fig. 4A, left, we detected PAPP-A expression in the invading trophoblast cells at the border of the junctional zone and decidua in D13 placentas of pregnant HF mice. This expression of PAPP-A is attenuated in the trophoblast cells of pregnant HF + DEHP mice. Quantitation by ImageJ analysis revealed a significant decline of PAPP-A immunostaining in HF + DEHP placentas compared to HF placentas (Fig. 4A, right). As in the case of PAPP-A, we observed decreased MUC1 expression in the labyrinth of the HF + DEHP placentas compared to HF placentas (Fig. 4B, *p < 0.05). MUC1 immunostaining was not affected in placentas of mice fed either normal diet or normal diet with exposure to DEHP (Supplementary Fig. S4). These results indicated that mice treated with DEHP and fed a HF diet alters the expression of PPARγ target genes in the placenta.

**Figure 5:**
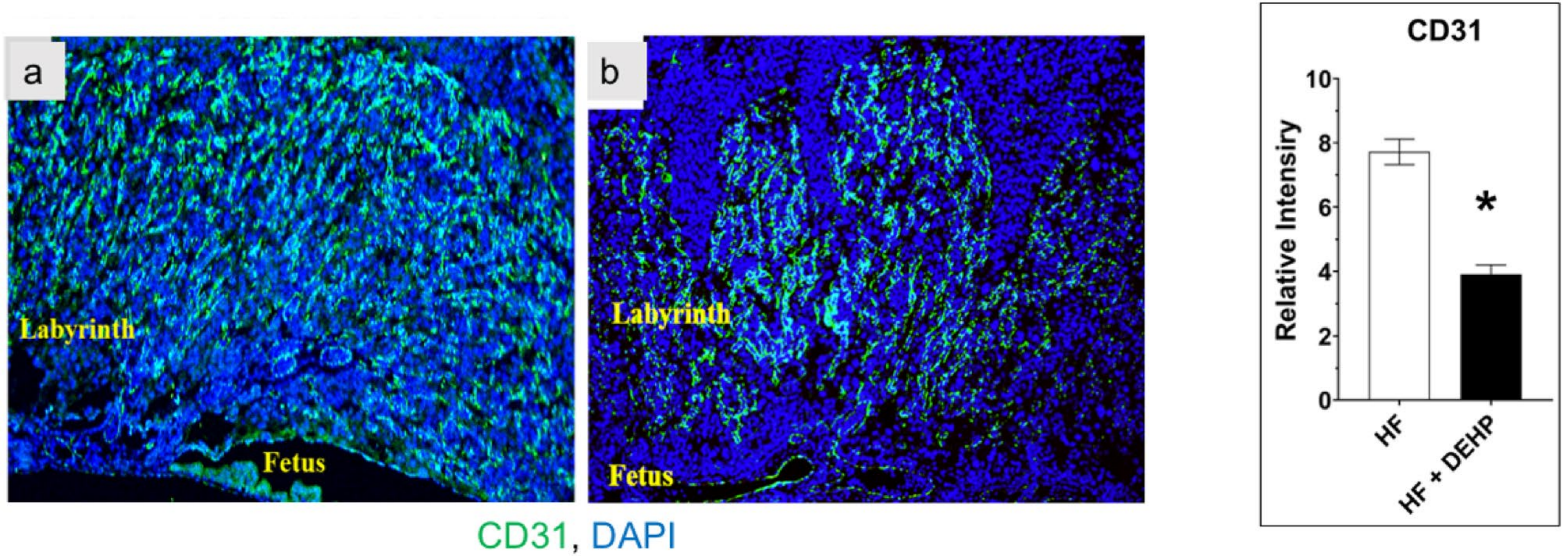
DEHP exposure and HF diet impacts vascularization of the placenta. Left. CD31 immunostaining of placental labyrinth from HF (panel **a**) and HF + DEHP (**b**) animals on D13 of pregnancy (10×) are shown. Green indicates immunostaining with CD31 and blue indicates DAPI. Right. ImageJ analysis of CD31 positive cells. The values represent mean ± SEM of five independent samples. Asterisks indicate statistically significant differences *p < 0.05.

The decline in MUC1 expression in HF + DEHP placentas raises the possibility that placental vascularization might be affected in mice by exposure to DEHP and fed a HF diet. As shown in Fig. 5, the placentas from HF mice exhibited abundant expression of CD31, a marker of endothelial cells, indicating a widespread vascular network in the labyrinth (left, panel a). The expression of CD31 in the labyrinth was severely attenuated in HF + DEHP placentas compared to HF placentas, indicating a defect in vascularization (Fig. 5, *p < 0.05). CD31 immunostaining was not affected in placentas of mice fed either normal diet or normal diet with exposure to DEHP (Supplementary Fig. S5). Collectively, these results indicated that DEHP alters key placental functions, presumably by altering PPARγ activity, which leads to downregulation of critical downstream pathways, such as PAPP-A and Muc1, controlled by this nuclear receptor.

**Figure 6:**
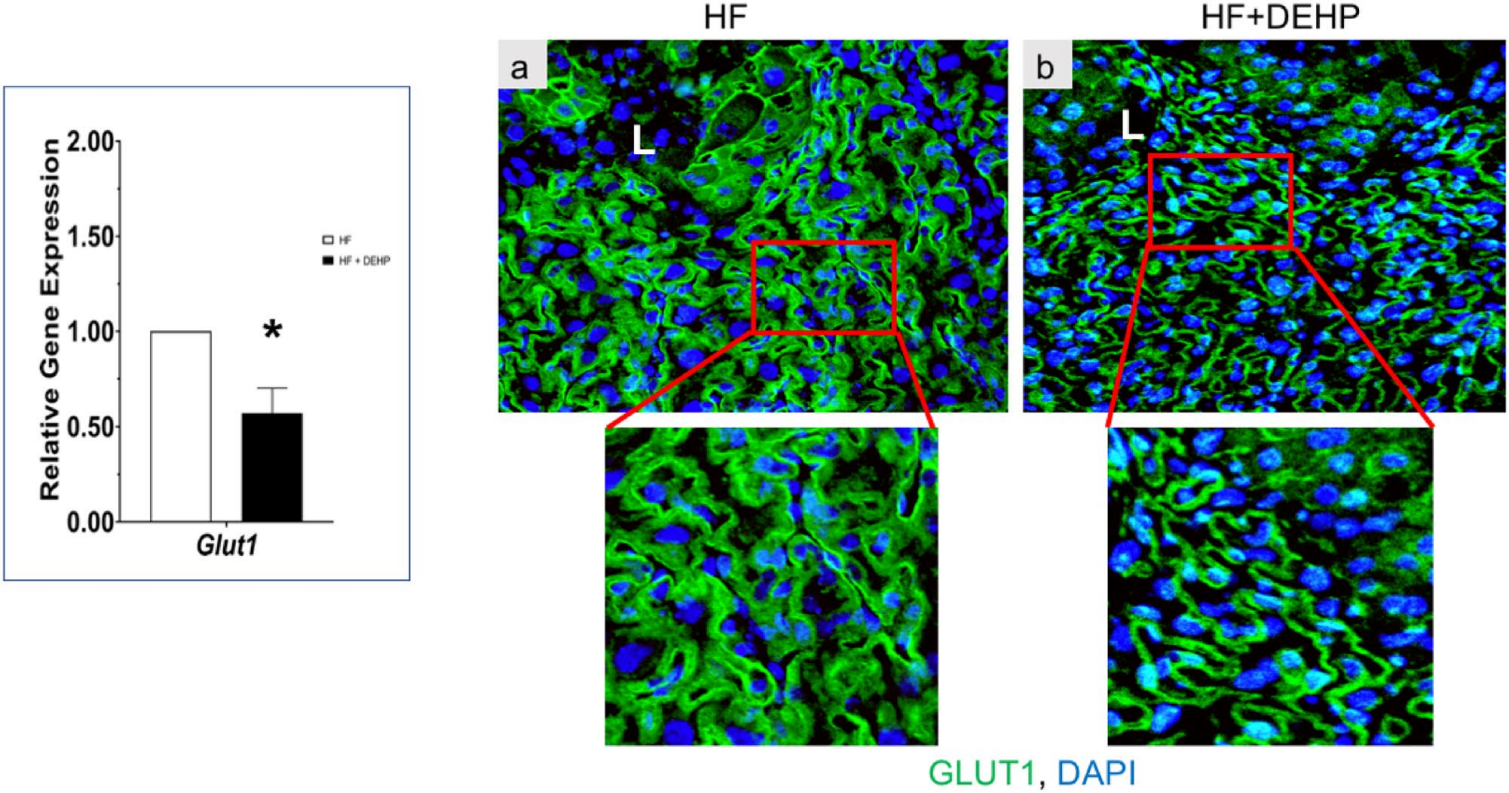
GLUT1 expression is attenuated in trophoblast cells in response to DEHP and HF diet. Left. Expression of *Glut1* in placental labyrinth from HF and HF + DEHP animals. Total RNA was isolated from the placenta on D13 of pregnancy and qPCR analysis was performed using primers specific for *Glut1*. Data represent mean ± SEM from four separate samples. Asterisks indicate statistically significant differences *p < 0.05. Right. GLUT1 immunostaining of placental labyrinth from HF (panel **a**) and HF + DEHP (**b**) animals on D16 of pregnancy are shown (20×). Lower panels indicate magnified images of the boxed areas. Green indicates immunostaining with GLUT1 and blue indicates DAPI. N = 5 for each group and representative images are shown.

Glucose is a critical nutrient for maintenance of fetal development and growth and its utilization is dependent on placental morphology, blood flow, and vascularity. Since placental vascularity was affected by exposure to DEHP and a HF diet, we considered the possibility that glucose transport could also be affected by these exposures. Previous studies have shown that PPAR_γ_ enhances glucose uptake in certain cells (such as adipocytes) by regulating the expression of glucose transporter 1 (Glut1)^42^. We, therefore, investigated the expression of *Glut1* transcripts in the placentas of HF and HF + DEHP mice and found its expression to be significantly downregulated in HF + DEHP animals (Fig. 6, left, *p < 0.05). We also investigated the expression of GLUT1 protein in HF and HF + DEHP placentas. As shown in Fig. 6, right, panel a, GLUT1 is expressed at the apical and basal sides of the syncytiotrophoblast cells of the labyrinth in placentas of HF mice. In contrast, in HF + DEHP placentas, GLUT1 expression was significantly reduced in the cytoplasm and plasma membrane of these cells (Fig. 6 panel b). Collectively, these results indicated that exposure of HF diet-fed pregnant mice to DEHP impairs GLUT1 expression in trophoblast cells, potentially compromising proper nutrient transport to the fetus and causing restriction of intrauterine growth.

## Discussion

A previous study by Toft et al*.* reported on the impact of phthalates on pregnancy outcome in humans^43^. It indicated that exposure to phthalates is associated with decreased pregnancy rates, high miscarriage rates, and preeclampsia in women. In this study, phthalate exposure was assessed by determining the concentrations of urinary phthalate metabolites, and pregnancy loss was assessed by clinical information on spontaneous abortions and/or urinary levels of human chorionic gonadotropin. Additional epidemiological studies also indicated that phthalate exposure is associated with a higher occurrence of pregnancy loss in women^44,45^. Surprisingly, in another study, Jukic et al. reported that DEHP metabolites were not associated with early pregnancy loss in humans^46^. While the reasons for the discrepancy in these results are unknown, there is a clear need to gain a better understanding of the effects of phthalates on pregnancy by using an appropriate animal model. An earlier study indicated that phthalate exposure of mice during early pregnancy leads to low birth weight and pregnancy loss^24^. However, this study was performed using a dose of DEHP (500 mg/kg/day), which far exceeds the level of environmental exposure (20 µg/kg/day). The relevance of these findings is therefore called into question, particularly due to our observation in the present study that mice raised on normal diet and exposed to an environmentally relevant level of DEHP during pregnancy did not exhibit any significant alteration in pregnancy outcome or placental morphology (Figs. 1, 2). Our study, however, revealed that exposure of pregnant mice to the same low dose of DEHP (20 µg/kg/day) can lead to an impairment in placental development and delivery of low birth weight pups if these mice are fed a HF diet. While the HF diet contains red dye 40 which exerts adverse effects in high doses, it is unlikely that the negligible amount of red dye 40 present in the HF diet used in this study would cause any harmful effects. However, we cannot completely rule out the possibility that red dye 40, rather than the fat, is synergizing with the phthalate. Nonetheless, the findings from this study suggest that maternal diet during pregnancy is a critical factor that determines whether an exposure to an environmental toxicant during pregnancy results in impaired placental and fetal development.

In mammals, the major determinant of intrauterine growth is nutrient delivery to the fetus via the placenta, which occurs primarily by diffusion and transporter-mediated mechanisms. Transport of nutrients in the placenta is dependent on placental size, morphology, transporter capacity/availability, and placental blood flow. Therefore, abnormal placental development is associated with impaired intrauterine growth. Analysis of placentas of pregnant mice fed a HF diet clearly indicated a disruption in placental architecture in response to DEHP exposure. Spongiotrophoblast cells, which normally reside in the junctional zone of the placenta and are marked by TPBPA expression, are aberrantly localized in the underlying labyrinth. The cyclin-dependent kinase inhibitor p57^kip2^, which plays an important role in the proper trophoblast differentiation and development of spongiotrophoblasts and labyrinth layer of placenta^31^, exhibited significantly decreased expression in DEHP-exposed HF placentas. This observation indicated that the programmed differentiation of trophoblast cells is derailed, causing disruption in placental architecture. Indeed, abnormal trophoblast differentiation and function is the basis of many placenta-based pregnancy disorders, including fetal growth restriction. It is, therefore, not surprising that a significant reduction in fetal weight was observed when mice fed a HF diet were exposed to DEHP.

We report that the impairment in trophoblast differentiation resulting from the combined effects of DEHP exposure and a HF diet during pregnancy is associated with an alteration in the signaling pathways controlled by PPARγ. A previous study reported that phthalates and their metabolites modulate the activity of PPARγ, a ligand-activated transcription factor, presumably by acting as agonist/antagonist ligands^47^. It is well-documented that PPARγ plays essential roles in placental development. PPARγ-null embryos die in mid-gestation due to placental abnormalities characterized by a small labyrinth, reduced spongiotrophoblasts, and expanded giant cell layers^35,37^. PPARγ regulates differentiation of labyrinthine trophoblast lineages, which, along with fetal endothelium, form the vascular exchange interface with maternal blood. Gcm1, a downstream target of PPARγ, has emerged as a potential mediator of its function because it regulates differentiation of syncytiotrophoblast SynT-II cells of the labyrinth^34,35^. Mice lacking *Gcm1* die at embryonic day 10.5 due to the absence of the placental labyrinth^48^. Indeed, studies have shown that PPARγ regulates labyrinthine differentiation, at least in part, through Gcm1^35,49,50^. A deficiency of PPARγ in mouse trophoblast stem cells was also shown to affect labyrinth cell lineages with a concurrent decrease in Gcm1^35^. Further studies have shown that treatment of trophoblast stem cells with rosiglitazone, a PPARγ agonist, led to induction of Gcm1 expression^35^. Rosiglitazone, however, did not alter the expression of syncytin A, a marker of SynT-I cells, and Ctsq, a marker of sinusoidal trophoblast giant cells in the labyrinth^35^. Interestingly, our results showed that *Gcm1* expression is significantly downregulated in placentas of HF + DEHP mice, while expressions of syncytin A and Ctsq remain unaffected. It is clear that DEHP + HF treatment did not affect the expression of PPARγ but impaired the expression of downstream pathways regulated by PPARγ. These results are consistent with our hypothesis that DEHP or its metabolites acts as potential regulators of transcriptional activity of PPARγ in the placenta, thereby interfering with the expression of PPARγ target genes and affecting labyrinthine differentiation. Use of chromatin immunoprecipitation (ChIP) in placental tissues exposed to diets and DEHP will enable us to validate the effects of these treatments on binding of endogenous PPARγ to relevant promoters, such as Gcm1.

Our study indicated that DEHP + HF diet exposure suppresses the expression of key PPARγ target genes which mediate placental functions. PAPP-A and MUC1 are well known transcriptional targets of PPARγ in placenta^40,51^. PAPP-A is a metalloproteinase that cleaves IGF binding proteins (IGFBPs) produced in the maternal decidua, and thereby increases the bioavailability of growth factors necessary to promote feto-placental growth^41^. Previous studies have also shown that PAPP-A regulates invasion of extravillous cytotrophoblasts in human placenta. Indeed, PAPP-A has long been recognized as a marker of fetal genetic disorders and adverse pregnancy outcomes^52,53^. The present study revealed a marked downregulation of PAPP-A protein in the junctional trophoblast layer of DEHP + HF placenta. Thus, it is conceivable that the invasive property of the trophoblast cells, which is critical for invasion into the uterine vasculature and pregnancy maintenance, is compromised when pregnant mice fed on HF diet are exposed to low levels of DEHP.

PPARγ function in the placenta is also mediated by its downstream target Muc1, which is expressed at the apical surface of the labyrinthine trophoblast around maternal blood sinuses^40,54^. MUC1 protein is involved in trophoblast adhesion to uterine endothelial cells and in trophoblast transendothelial migration^55^. Our investigation indicated that exposure to DEHP + HF downregulates the expression of MUC1 in labyrinthine trophoblast cells of HF diet-fed pregnant mice. Indeed, CD31 staining indicated that vascularization in the labyrinth is markedly suppressed in response to this exposure. Disruption of labyrinth vascularization, which results in nutrient deprivation in the fetus, is one of the main causes of intrauterine growth restriction. Glucose is a major nutrient which is transported across the placenta by glucose transporters. The glucose transporter GLUT1 is the primary isoform involved in the transplacental movement of glucose. Our studies revealed downregulation of *Glut1* mRNA and defective localization of GLUT1 protein in trophoblast cells of HF + DEHP placentas. In HF placentas, GLUT1 protein was localized to the plasma membrane and cytoplasm of the trophoblast cells present in the labyrinth. In HF + DEHP placentas, GLUT1 protein levels were markedly reduced in the trophoblast cells. Thus, the combined effects of an exposure to DEHP and a HF diet during pregnancy suppress the expression of the GLUT1 protein. It is possible that this deficiency in the glucose transporter expression dampens the transfer of glucose from mother to fetus, affecting fetal growth. While the underlying mechanism of the DEHP-mediated defect in GLUT1 expression remains unclear, alterations in cellular glucose metabolism combined with decreased vascularization in the labyrinth, are likely causes of growth retardation in pups born to HF + DEHP dams.

In summary, our study has revealed that exposure to an environmentally relevant dose of DEHP, which is deemed safe, can have a detrimental effect on developing placental vasculature and fetal growth when accompanied by maternal ingestion of a HF diet during pregnancy. This, in part, is due to the dysregulated transcriptional function of PPAR_γ_ which likely arises from the synergistic effects of DEHP exposure and a HF diet. These results provide novel insight into potential etiological factors underlying pathological placentation, including IUGR, which are among the leading causes of maternal and fetal morbidity and mortality.

## Supplementary Information

Supplementary Information.
