# Supplementary material for: Maternal high-fat diet during pregnancy with concurrent phthalate exposure leads to abnormal placentation
Source: Sci Rep. 2021 Aug 16;11:16602. doi: 10.1038/s41598-021-95898-4 (PMC8368193; doi:10.1038/s41598-021-95898-4)
Supplement: Supplementary file 1 — Supplementary Information. [file 41598_2021_95898_MOESM1_ESM.pdf]

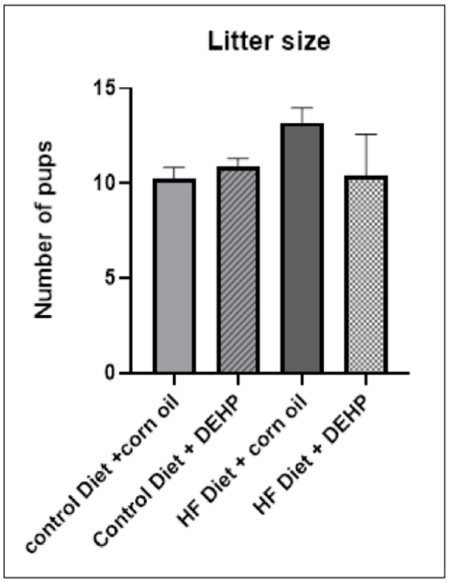

Fig. S1

Figure S1: Exposure to DEHP and a HF diet during pregnancy does not affect litter size. Female mice from day 1 of pregnancy were fed normal diet or ND (control) or normal diet and exposed to 20 µg/kg/day of DEHP (DEHP), HF diet (HF) or HF diet and exposed to 20 µg/kg/day of DEHP (HF+DEHP) as described in the Materials and Methods. Litter size (live and dead pups) was determined at birth. Data are represented as the mean of pups pooled from 8 animals in each group.

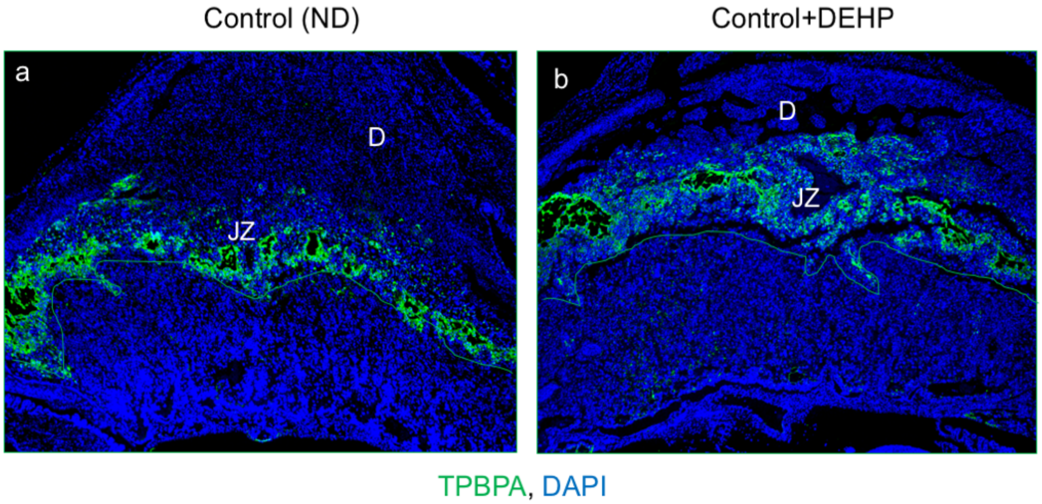

Fig. S2

Figure S2: Placental architecture is unaffected in mice exposed to DEHP and fed a normal diet. Female mice from day 1 of pregnancy were fed normal diet (control) or normal diet and exposed to 20 µg/kg/day of DEHP (DEHP) as described in the Materials and Methods. Placental sections from control (panel a) and DEHP (panel b) mice on D13 of pregnancy were subjected to immunofluorescence using trophoblast specific protein alpha (TPBPA) antibody. TPBPA positive spongiotrophoblast layer is demarcated by a white line. JZ: junctional zone, D: decidua. N=4 for each group and representative images are shown.

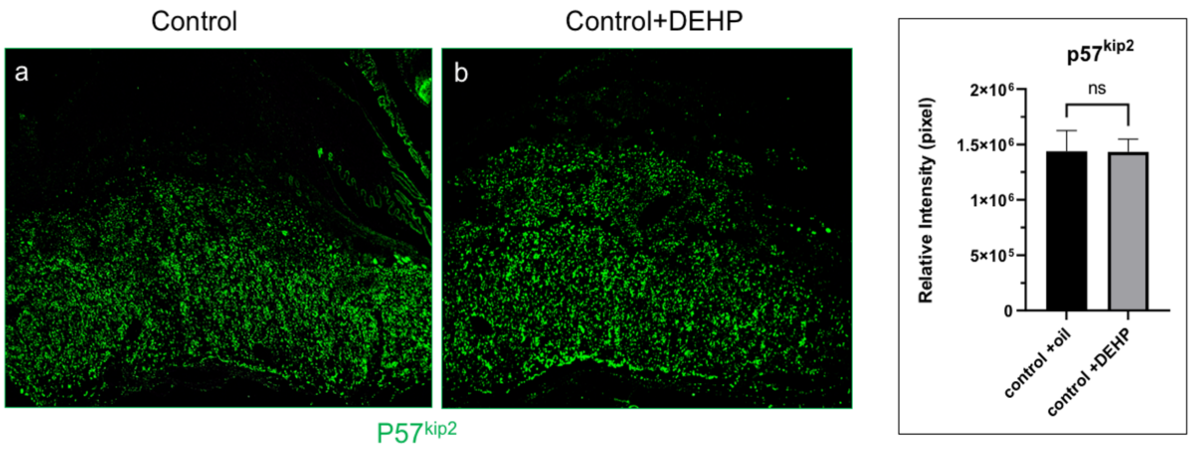

Fig. S3

Figure S3: DEHP exposure does not affect trophoblast differentiation. Left. Placental sections from control (panel a) and DEHP (panel b) mice on D13 of pregnancy were subjected to immunofluorescence using P57Kip2 antibody. N=4 for each group and representative images are shown. **Right.** ImageJ analysis of P57Kip2 positive cells. The values represent mean ± SEM of four independent samples.

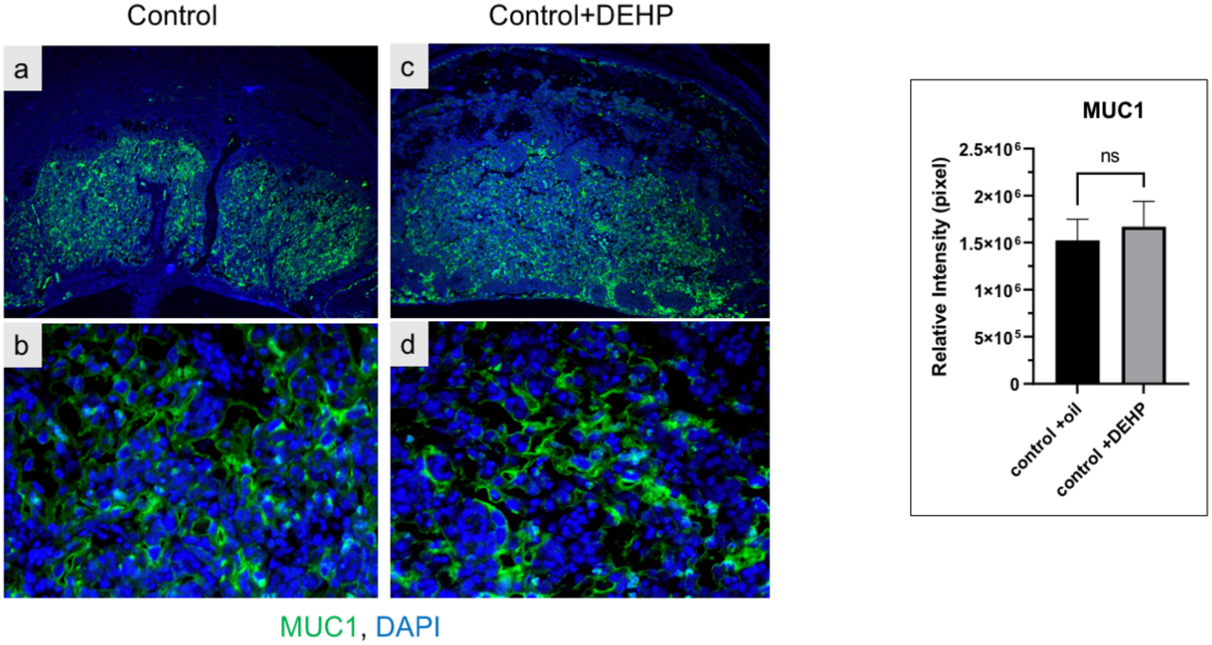

Fig. S4

Figure S4: Exposure to DEHP does not affect the expression of MUC1. Left. Placental sections from control (panels a and b) and DEHP (panels c and d) mice on D13 of pregnancy were subjected to immunofluorescence using MUC1 antibody. Panels b and d represent magnified images of trophoblast cells from control and DEHP placentas (4X). **Right.** Immuno-positive cells for MUC1 were analyzed by ImageJ software. The values represent mean ± SEM of four independent samples.

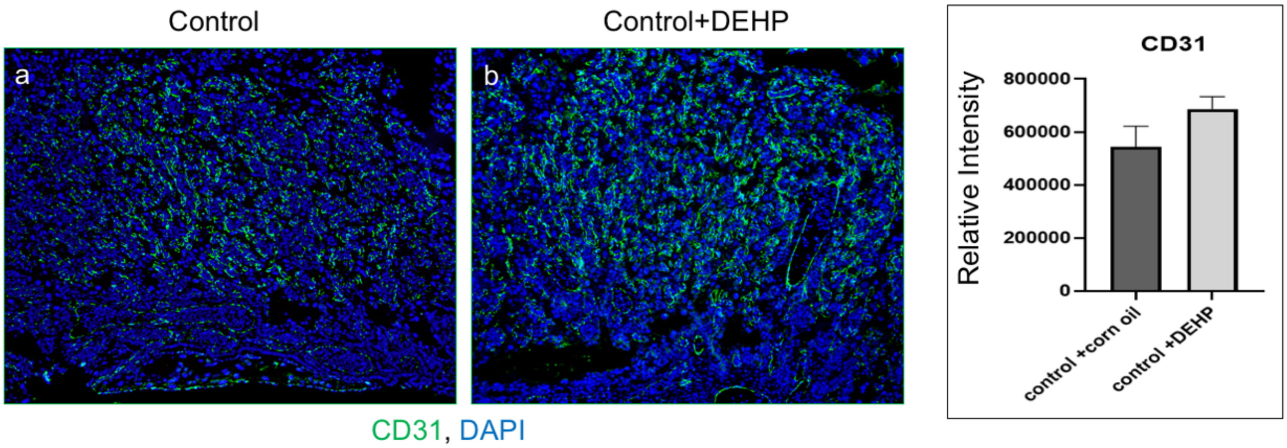

Fig. S5

Figure S5: Exposure to DEHP does not affect the expression of CD31. Left. Placental sections from control (panel a) and DEHP (panel b) mice on D13 of pregnancy were subjected to immunofluorescence using CD31 antibody. N=4 for each group and representative images are shown. **Right.** ImageJ analysis of CD31 positive cells. The values represent mean ± SEM of four independent samples.
